# Release and uptake of volatile organic compounds by human hepatocellular carcinoma cells (HepG2) *in vitro*

**DOI:** 10.1186/1475-2867-13-72

**Published:** 2013-07-17

**Authors:** Paweł Mochalski, Andreas Sponring, Julian King, Karl Unterkofler, Jakob Troppmair, Anton Amann

**Affiliations:** 1 Breath Research Institute, Austrian Academy of Sciences, Rathausplatz 4, A-6850 Dornbirn, Austria; 2 Univ.-Clinic for Anesthesia, Innsbruck Medical University, Anichstr, 35, A-6020 Innsbruck, Austria; 3 Vorarlberg University of Applied Sciences, Hochschulstr. 1, A-6850 Dornbirn, Austria; 4 Daniel-Swarovski Research Laboratory, Department of Visceral-, Transplant- and Thoracic Surgery, Innsbruck Medical University, Innrain 66, A-6020 Innsbruck, Austria

**Keywords:** HepG2 cells, Volatile organic compounds, VOCs, Biomarkers, GC-MS, Emission of metabolites, Enzymes expression

## Abstract

**Background:**

Volatile organic compounds (VOCs) emitted by human body offer a unique insight into biochemical processes ongoing in healthy and diseased human organisms. Unfortunately, in many cases their origin and metabolic fate have not been yet elucidated in sufficient depth, thus limiting their clinical application. The primary goal of this work was to identify and quantify volatile organic compounds being released or metabolized by HepG2 hepatocellular carcinoma cells.

**Methods:**

The hepatocellular carcinoma cells were incubated in specially designed head-space 1-L glass bottles sealed for 24 hours prior to measurements. Identification and quantification of volatiles released and consumed by cells under study were performed by gas chromatography with mass spectrometric detection (GC-MS) coupled with head-space needle trap device extraction (HS-NTD) as the pre-concentration technique. Most of the compounds were identified both by spectral library match as well as retention time comparison based on standards.

**Results:**

A total of nine compounds were found to be metabolised and further twelve released by the cells under study (Wilcoxon signed-rank test, p<0.05). The former group comprised 6 aldehydes (2-methyl 2-propenal, 2-methyl propanal, 2-ethylacrolein, 3-methyl butanal, n-hexanal and benzaldehyde), n-propyl propionate, n-butyl acetate, and isoprene. Amongst the released species there were five ketones (2-pentanone, 3-heptanone, 2-heptanone, 3-octanone, 2-nonanone), five volatile sulphur compounds (dimethyl sulfide, ethyl methyl sulfide, 3-methyl thiophene, 2-methyl-1-(methylthio)- propane and 2-methyl-5-(methylthio) furan), n-propyl acetate, and 2-heptene.

**Conclusions:**

The emission and uptake of the aforementioned VOCs may reflect the activity of abundant liver enzymes and support the potential of VOC analysis for the assessment of enzymes function.

## Background

Volatile organic compounds (VOCs) emitted by the human body have a great potential for medical diagnosis and therapeutic monitoring [1-5]. Their analysis offers a unique insight into biochemical processes ongoing in healthy and diseased human organisms. Breath analysis holds a distinguished status in this context as it is non-invasive and breath biomarkers can provide valuable information on disease processes, or metabolic disorders occurring even in distant parts of the body. For instance, volatile compound profiles were shown to be different in lung cancer patients as compared to healthy controls [6-9] and proved to be useful in the quantification of oxidative stress [10,11]. Unfortunately, the origin and metabolic fate of numerous breath VOCs have not been elucidated in sufficient depth, thereby limiting the clinical application of breath tests. In this context, the knowledge of precise biochemical pathways of volatile compound formation or at least the information if a compound is produced in human cells (both normal cells or cancerogenous cells), emitted by bacteria in the gut, or released by pathogenic organisms (e.g., bacteria, fungi) is highly desirable. For example, over the last few years an effort was made to pinpoint VOCs emitted specifically by cancer cells [12-16], bacteria [17-19], or fungi [5].

HepG2 liver cells are of particular interest in this context: volatile compounds released by the liver might be interesting biomarkers related to the activity of various enzymes including those involved in drug metabolism (such as cytochrome P450 enzymes). Here we investigate one of the most frequently studied liver cell lines, HepG2. This cell line has been derived from a 15 year old male patient with liver carcinoma. HepG2 cells possess epithelial morphology and secrete a variety of major plasma proteins (e.g., albumin, transferrin and the acute phase proteins fibrinogen, alpha 2-macroglobulin, alpha 1-antitrypsin, transferrin, and plasminogen). These cells can be grown successfully in large scale cultivation systems. Work by Castaneda et al. [20] established the production of undecane-2-one in HepG2 cells exposed to ethanol. However, a detailed GC-MS-based investigation of the release or uptake of volatile compounds by HepG2 cells is still lacking. Hence, the primary goal of this work was to identify volatile organic compounds released or metabolized by HepG2 hepatocellular carcinoma cells. For this purpose an experimental setup combining head-space needle trap extraction (NTD) and gas chromatography – mass spectrometry (GC-MS) was applied. GC-MS is the gold standard in the analysis of volatile compounds. The majority of compounds (with few exceptions) were identified not only by spectral library match, but also by comparison of retention times using native standards.

## Results and discussion

### Method validation

Limits of detection (LODs) were calculated using the mean value of the blank responses and their standard deviations obtained on the basis of 10 blank measurements [21]. The LOD values ranged from 0.01 ppb for 3-methyl thiophene to 0.3 ppb for 2-methyl propanal (see Table 1). The relative standard deviations (RSDs) were calculated on the basis of five consecutive analyses of standard mixtures. The calculated RSDs varied from 2.5-12%, which is adequate for the aims of this study. The system response was found to be linear within the investigated concentration ranges, as shown in Table 1, with the coefficients of variation ranging from 0.974 to 0.999.

**Table 1:** Retention times, quantifier ions, LODs, RSDs, coefficients of variation and linear ranges of compounds under study

**VOC**	**CAS**	**R**_**t **_**[min]**	**Quantifier ion**	**LOD [ppb]**	**RSD [%]**	**R**^**2**^	**linear range [ppb]**
Dimethyl sulfide	75-18-3	16.35	62	0.08	6	0.997	0.24-70
Isoprene	78-79-5	18.18	67	0.04	4.5	0.999	0.12-12
2-Propenal, 2-methyl-	78-85-3	19.11	70	0.03	8	0.993	0.1-12
Propanal, 2-methyl-	78-84-2	19.43	72	0.3	9	0.977	0.9-150
Sulfide, ethyl methyl	624-89-5	20.84	61	0.02	6	0.999	0.06-5.3
2-Ethylacrolein	922-63-4	23.19	Not quantified				
Butanal, 3-methyl-	590-86-3	23.48	44	0.14	9	0.978	0.4-350
Butanal, 2-methyl-	96-17-3	23.53	Not quantified, RT confirmed				
2-Pentanone	107-87-9	24.10	43	0.05	7	0.998	0.15-9
n-Propyl acetate	109-60-4	24.88	43	0.06	3	0.998	0.18-9
Thiophene, 3-methyl-	616-44-4	26.02	97	0.01	3	0.998	0.03-9.6
2-Heptene	14686-13-6	25.11	Not quantified				
Propane, 2-methyl-1-(methylthio)-	5008-69-5	27.48	Not quantified				
n-Hexanal	66-25-1	27.83	56	0.2	9	0.994	0.6-15
n-Propyl propionate	106-36-5	28.11	75	0.03	10	0.996	0.1-7
n-Butyl acetate	123-86-4	28.27	56	0.04	10	0.997	0.12-8
3-Heptanone	106-35-4	30.60	85	0.03	2.5	0.997	0.09-7
2-Heptanone	110-43-0	30.78	43	0.03	7	0.998	0.09-6.5
Benzaldehyde	100-52-7	30.99	106	0.05	12	0.998	0.15-12
Furan, 2-methyl-5-(methylthio)-	13678-59-6	31.05	128	0.03	7	0.988	0.09-8
3-Octanone	106-68-3	33.47	99	0.1	7	0.981	0.3-5.5
2-Nonanone	821-55-6	36.25	58	0.07	11	0.974	0.21-5.7

### HepG2 cells

The total number of the HepG2 cells and their viability after 24 hours of incubation in the sealed measurement bottles is presented in Table 2. Cell numbers ranged from 2.6×10^6^ to 30.7×10^6^ (mean 17.9×10^6^), whereas the viability varied from 80.1 % to 99.6% (mean 91.1%). The applied experimental conditions thus did not significantly affect the viability of cells and it can consequently be assumed that the released and consumed species reflect the normal metabolism of cells under study.

**Table 2:** Total number of cells, number of living cells and viability at the end of the cultivation

**Culture**	**Total number of cells ×10**^**6**^	**Number of living cells ×10**^**6**^	**Viability [%]**
1	8.47	6.90	81.4
2	10.17	8.15	80.1
3	2.60	2.50	96.1
4	28.5	28.4	99.6
5	30.7	27.6	89.9
6	26.8	26.7	99.6
mean	**17.9**	**16.7**	**91.1**

### Uptake of VOCs by HepG2 cells

The uptake of volatiles by HepG2 cells from the culture medium was a matter of interest, as it can give valuable insights into the metabolism of the cells under study. A total of nine compounds were found to be metabolised in the HepG2 cell cultures as compared to blank samples (Wilcoxon signed-rank test, p < 0.05). The detection and quantification incidences as well as the observed concentration ranges in media and cell cultures are given in Table 3. The majority of them were aldehydes including the following six representatives: 2-methyl 2-propenal, 2-methyl propanal, 2-ethylacrolein, 3-methyl butanal, n-hexanal and benzaldehyde. Apart from these there were two esters (n-butyl acetate and n-propyl propionate) and the hydrocarbon isoprene. In the case of 2-ethylacrolein the identification was based exclusively on the NIST mass spectral library match and the Wilcoxon test was performed using uncalibrated peak areas. The levels of 2-methyl butanal were also found to decrease, however, a proper integration and quantification of this compound was not possible due to the poor separation from 3-methyl butanal and the absence of unique ions that could be used for these purposes. Interestingly, saturated aldehydes were taken up more readily than the unsaturated ones. For example, the headspace concentrations of 2-methyl propanal and 3-methyl butanal dropped by over 98% after 24 hours of incubation, whereas the corresponding drop for 2-methyl 2-propenal and 2-ethylacrolein amounted to only 60% and 75%, respectively.

**Table 3:** **Detection (n**_**d**_**) and quantification (n**_**q**_**) incidences, concentration ranges and medians in the headspace of medium and cell cultures**

		**Experiment No**						**Incidence n**_**d**_**(n**_**q**_**)**	**Range (median)**	**Experiment No**						**Incidence n**_**d**_**(n**_**q**_**)**	**Range (median)**
		1	2	3	4	5	6			1	2	3	4	5	6		
**Uptake**	Isoprene	2.1	2.6	0.9	8.5	2.2	1.6	6(6)	0.9-8.5(2.2)	7.6	11.9	1.7	18	9.3	13.5	6(6)	1.7-18(10.5)
	2-Propenal, 2-methyl-	0.26	0.21	0.28	0.30	0.43	0.41	6(6)	0.21-0.43(0.29)	1.0	0.62	0.85	2.07	0.71	0.68	6(6)	0.58-2.07 (0.73)
	Propanal, 2-methyl-	0.87	0.92	0.9	0.96	1.18	1.10	6(4)	0.9-1.18(0.94)	42.5	50.9	32.5	160	47.4	54.4	6(6)	32.5-160(49)
	*2-Ethylacrolein*	*1900*	*2900*	*2930*	*1940*	*1570*	*1580*	*6*	*1574-2923(1937)*	*16000*	*19500*	*4760*	*10900*	*4880*	*5100*	*6*	*4758-19500 (8020)*
	Butanal, 3-methyl-	0.8	1.07	n.d.	1.48	n.d.	n.d.	3(3)	0.8-1.48(1.07)	70.5	79.8	51	399	122	126	6(6)	51-399(100)
	n-Hexanal	1.3	0.9	1.0	0.56	0.50	0.51	6(6)	0.5-1.3(0.74)	11	12.8	6.0	11	2.7	3.0	6(6)	2.7-13(8.5)
	n-Propyl propionate	0.15	1.46	1.38	n.q.	n.d.	n.d.	4(3)	0.15-1.46(1.0)	5.37	5.2	3	3.84	4.17	3.68	6(6)	3.7-5.4(4.1)
	n-Butyl acetate	0.3	0.52	0.28	2.9	0.17	0.2	6(6)	0.17-2.9(0.3)	6.5	9.8	2.3	4.8	1.47	2.12	6(6)	1.5-9.8(3.4)
	Benzaldehyde	0.31	0.27	0.6	0.29	0.31	0.3	6(6)	0.27-0.6(0.3)	1.5	1.63	1.57	13.8	3.14	2.96	6(6)	1.5-14(2.4)
**Release**	Dimethyl sulfide	12.8	12.4	7.5	80	69	77	6(6)	7.5-80.3(43)	9	8.2	4	8	9.5	10.9	6(6)	4-11(8.6)
	Sulfide, ethyl methyl	0.11	0.12	n.q.	0.29	0.26	0.23	6(5)	0.11-0.29(0.23)	n.d.	n.d.	n.d.	n.d.	n.d.	n.d.	0(0)	n.d.
	2-Pentanone	3.3	4	1.8	3.6	4.0	4.0	6(6)	1.8-4.0(3.8)	0.18	0.2	0.15	0.7	0.65	0.7	6(5)	0.15-0.7(0.45)
	n-Propyl acetate	0.59	0.62	0.22	0.27	0.32	0.22	6(6)	0.22-0.62(0.33)	n.d.	n.d.	n.q.	n.q.	n.q.	n.q.	4(0)	n.q.
	Thiophene, 3-methyl-	0.07	0.09	0.03	0.12	0.16	0.12	6(6)	0.03-0.16(0.11)	n.q.	n.q.	n.q.	0.03	0.05	0.04	6(3)	0.03-0.05(0.04)
	*2-Heptene*	*2500*	*4200*	*3700*	*2700*	*2600*	*1500*	*6*	*1512-4170(2680)*	*680*	*710*	*950*	*500*	*600*	*480*	*6*	*500-950(600)*
	*Propane, 2-methyl-1-(methylthio)-*	*650*	*580*	*350*	*6400*	*5900*	*5800*	*6*	*346-6400(3200)*	*-*	*-*	*-*	*-*	*-*	*-*	*0*	*-*
	3-Heptanone	0.23	0.89	0.36	0.2	0.2	0.14	6(6)	0.13-0.9(0.22)	0.14	0.17	0.12	0.09	0.09	0.08	6(6)	0.09-0.17(0.1)
	2-Heptanone	0.36	0.44	0.19	0.76	0.66	0.67	6(6)	0.19-0.76(0.55)	n.q.	n.q.	n.q.	0.4	0.09	0.23	6(3)	0.09-0.4(0.25)
	Furan, 2-methyl-5-(methylthio)-	6.4	6.9	2	2.4	2.1	2.0	6(6)	2.0-6.9(2.3)	n.d.	n.d.	n.d.	n.d.	n.d.	n.d.	0(0)	n.d.
	3-Octanone	0.82	0.92	1.03	0.37	0.36	0.39	6(6)	0.36-1.03(0.6)	n.d.	n.d.	n.d.	n.d.	n.d.	n.d.	0(0)	n.d.
	2-Nonanone	3.2	3.6	1.8	1.8	1.7	1.8	6(6)	1.73-3.56(1.8)	n.q.	n.q.	n.q.	n.q.	n.q.	n.q.	6(0)	n.q.

Compounds in italics were not quantified for reasons mentioned in the text, n.d. – not detected (<LOD), n.q. - not quantified (<LOQ).

Although frequently the origin and metabolic fate of volatile organic compounds in human organism remain ambiguous, several biochemical pathways could explain the uptake and release of species by HepG2 cells. Aldehydes can be irreversibly oxidized by aldehyde dehydrogenases (ALDHs) to their corresponding carboxylic acids (e.g., acetaldehyde into acetate, hexanal into hexanoate), or reduced to alcohols by alcohol dehydrogenases (ADHs) [22]. Both ALDHs and ALHs are very abundant in human liver [22-24]. ALDHs catalyze the oxidation of a wide range of aromatic and aliphatic aldehydes, however, acetaldehyde is believed to be their most important substrate. Despite the fact that ADHs can also convert aldehydes into alcohols [25], their primary function in human liver seems to be the breakdown of alcohols (mainly ethanol) naturally contained in food [24]. Moreover, the expression of ALDHs and their enzymatic activity were also evidenced to be elevated in liver cancer cells [26]. Thus, oxidation by ALDHs appears to be the main reason of the uptake of the aldehydes observed within this study.

The activity of another abundant class of human liver enzymes, namely carboxylesterases (CESs) [27], can explain the observed uptake of two esters n-butyl acetate and n-propyl propionate. For example, CESs could catalyze the hydrolysis of n-butyl acetate into acetic acid and 1-butanol, which could be converted into n-butanal by ADHs and subsequently into butanoic acid by ALDHs. The aforementioned hypothesis is supported by the fact that lung cells (both cancer and normal) exhibiting also elevated CESs levels [27] were found to consume n-butyl acetate during *in vitro* studies [12,15].

The fivefold decrease of the isoprene levels in the HepG2 culture headspace is consistent with the previous experiments demonstrating the cytochrome P450 oxidation of this hydrocarbon to mono- and di-epoxides by human liver microsomes [28-31]. Thus, in human liver isoprene is metabolized mainly to 3,4-epoxy-3-methyl-1-butene and 3,4-epoxy-2-methyl-1-butene, which next are hydrolysed by epoxide hydrolase to vicinal diols (2-methyl-3-buten-1,2-diol and 3-methyl-3-buten-1,2-diol). Isoprene metabolism in the liver was also suggested by Miekisch et al. [32] on the basis of relatively low hepatic venous blood concentrations of this hydrocarbon in pigs. Isoprene is the major hydrocarbon produced in human organism exhibiting high abundances in breath (mean 100 ppb) and blood [32,33]. The most widespread hypothesis suggests that isoprene in humans is a by-product of cholesterol biosynthesis [4,33]. According to it isoprene is produced non-enzymatically by acid-catalyzed formation from dimethylallyl pyrophosphate (DMAPP). However, this reaction is rather slow at physiological pH and unlikely to completely explain the high isoprene levels in human organism [34,35]. Indeed, there is growing evidence provided by a number of recent studies suggesting that other metabolic sources may contribute to isoprene formation in the humans [32,33,36,37]. In this context it is interesting to note that the isoprene concentration in human breath increases approximately by a factor of 5 during exertion of effort on a stationary bicycle [37-42]. Isoprene might therefore not only be produced in the liver, but also in the muscles.

The studies on uptakes and releases of volatile organic compounds by human cell lines are relatively sparse. Consequently, it is difficult to relate findings obtained within this study to available literature data. Up to now only lung cells (both cancer and normal) received more widespread attention. Similar as in the case of HepG2 cells an uptake of aldehydes has been reported in cultures of human lung cancer cells [12,13,15]. This is not surprising as ALDHs are also highly expressed in lung cancer cells [43]. Conversely, Rutter et al. [44] demonstrated that the release of acetaldehyde by lung cancer cells was three times higher than by normal ones. However, this finding could be the result of different media composition (e.g. presence or absence of ethanol) and unequal increase of ADHs and ALDHs activity in cancer cells [45]. Nevertheless, a similar consumption has also been observed in cultures of normal lung cells [12]. Both cancer and normal lung cells were also shown to metabolise some esters (e.g. n-butyl acetate) during *in vitro* studies [12,15], which can be associated with the high expression of CESs in lung tissue [27].

### Release of VOCs by HepG2 cells

Twelve compounds were found to be released by HepG2 cells (see Table 3). The predominantly represented chemical classes within this group were ketones and volatile sulphur compounds (VSCs), both with five species (2-pentanone, 3-heptanone2-heptanone3-octanone, 2-nonanone, dimethyl sulfide, ethyl methyl sulfide, 3-methyl thiophene, 2-methyl-1-(methylthio)- propane, and 2-methyl-5-(methylthio) furan). There was also one hydrocarbon (2-heptene) and one ester (n-propyl acetate). Two compounds (2-heptene and 2-methyl-1-(methylthio)- propane) were not quantified due to the unavailability of pure substances from commercial vendors and their levels were assessed only on the basis of peak areas. The highest concentrations were observed for dimethyl sulfide (DMS; mean of 41 ppb in cell cultures vs. 8.6 ppb in media) and 2-pentanone (3.8 vs 0.45 ppb). The majority of the remaining species exhibited mean concentration values below 1 ppb after 24 hours of incubation.

A potential pathway leading to ketones production by HepG2 cells involves alcohol dehydrogenases (ADHs). ADHs are very abundant in liver and play a major role in hepatic ethanol metabolism [22,23,25,46]. They are also capable of metabolizing longer-chain and cyclic alcohols, however, primary alcohols seem to be their preferred substrates [22,23]. Although secondary alcohols were shown to be rather poor substrates for ADHs [23], catalysis of ketones production from secondary alcohols has been evidenced in the literature (e.g., acetone from 2-propanol, 2-octanone from 2-octanol) [23,25,47]. Moreover, the total alcohol dehydrogenases activity is significantly higher in liver cancer tissues than in healthy ones, significantly exceeding the activity of ALDHs [45,48]. Thus, all observed ketones can originate from the respective secondary alcohols. The source of the secondary alcohols remains unclear. Perhaps the applied medium contained small amounts of long-chain secondary alcohols. An alternative pathway leading to the formation of heavier ketones in humans is β-oxidation of branched-chain fatty acids. For example, 3-heptanone was found to be a product of valproic acid metabolism [3] and 4-heptanone was shown to originate from 2-ethylhexanoic acid [49]. The potential substrates for this metabolic pathway could in turn be metabolites of the respective branched-chain primary alcohols (e.g. 2-ethylhexanoic acid from 2-ethylhexanol). However, it is not clear if these substrates were present in the applied medium.

The second most dominant chemical class amongst the released species were volatile sulphur compounds (VSCs) with DMS as the most abundant analyte. The production of VSCs in humans is ascribed to the metabolization of the sulfur-containing amino acids methionine and cysteine in the transamination pathway [50]. In liver thiol S-methyltransferase forms methyl thioethers via the methylation of thiols [50-52]. For instance, DMS is formed via the methylation of methyl mercaptane [50]. Although production of methanethiol by L-methionine γ-lyase in humans is well documented, little is known about the formation of other thiols.

The origin of the two remaining species n-propyl acetate and 2-heptene remains unclear.

Whereas, human lung cells were reported to release some ketones (e.g. 2-pentanone, 2-hexanone, 2-octanone) [12,14], none sulphur species were observed to be liberated by these cells. This difference could be ascribed to the expression of liver-specific enzymes. Amongst hydrocarbons (HCs), only 2-heptene was found to be produced by HepG2. This finding clearly distinguishes HepG2 cells from lung cancer cells liberating numerous unsaturated and branched hydrocarbons [12,15]. Nevertheless, this chemical class is of particular interest as some HCs have been proposed as non-invasive markers of numerous diseases in the human organism [6-8] and their origin is still not clear. Interestingly, n-propyl acetate found to be emitted by HepG2 is also released by normal lung cells but not by cancer ones [12].

## Conclusions

The present study aimed at the identification and quantification of volatile organic compounds emitted or metabolized by HepG2 hepatocellular carcinoma cells. For this purpose gas chromatography with mass spectrometric detection coupled with head-space needle trap extraction (HS-NTD) as pre-concentration technique was applied. Nine species were found to be consumed and further 12 released by HepG2 cells. The emission and uptake of the aforementioned species may be explained by the activity of enzymes that are particularly abundant in human liver and additionally highly expressed in cancer cells. Thus, aldehydes were probably oxidized by aldehyde dehydrogenases to carboxylic acids, ketones were presumably the products of branched, or secondary alcohols metabolism and thioethers release could be an expression of thiol S-methyltransferase activity.

Several limitation of the study can be indicated. Firstly, the study involved transformed hepatocytes, which may exhibit an altered metabolism as compared to the normal ones [26,45]. Next, no additional liver-resident cells were included in the present study (e.g. representing other tissues of this complex organ). Thus, their contribution to the production and metabolism of VOCs in the liver remains to be established. Moreover, *in vivo* metabolic pathways may be regulated by numerous factors (e.g. hormones, ATP levels, oxygen concentration), which may have been lacking in our *in vitro* experiments. The initial background levels of VOCs were not strictly equal during cultivations. This results from the fact, that before each experiment fresh medium was prepared and purged, frequently from components originating from different production lots. These initial VOC levels could also be affected by small fluctuations of purging conditions (e.g. flow rate). Consequently, the production and consumption rates of VOCs under study did not depend exclusively on the number of cells and their metabolism.

In summary, the analysis of volatile organic compounds has the potential to identify and monitor enzyme activities. This feature may be helpful for the detection and analysis of cancers, which may carry mutations in metabolic enzymes [26,45,53].

## Methods

### Chemicals and calibration mixtures

Gaseous multi-compound calibration mixtures were prepared from pure liquid substances. The majority of them were purchased from Sigma-Aldrich (Austria); 2-methyl 2-propenal (95%), 2-methyl propanal (99.5%), ethyl methyl sulfide (95.5%), 3-methyl butanal (97%), 2-pentanone (99%), n-propyl acetate (98%), 3-methyl thiophene (98%), n-hexanal (98%), isoprene (99%) and 2-heptanone (98%). Moreover, dimethyl sulfide (99%), n-butyl acetate (99.7%), benzaldehyde (99%) and 2-methyl butanal (99%) were obtained from Fluka (Switzerland), whereas n-propyl propionate (98%) was provided by SAFC (USA). 3-Octanone (99%) was purchased from Acros Organic (Belgium), 3-heptanone (98%) from Alfa Aesar (USA), 2-methyl-5-(methylthio) furan (99%) from Chemos (Germany) and 2-nonanone (98%) from Merck Schuchardt (Germany).

Gaseous calibration mixtures were produced by means of a GasLab calibration mixtures generator (Breitfuss Messtechnik, Germany). The GasLab unit consists of an integrated zero air generator, a 2-stage dynamic injection module for evaporating a liquid and diluting it with zero air, and a humidification module enabling the preparation of gas mixtures at well-defined humidity levels (up to 100% relative humidity (RH) at 37°C). When using pure liquid substances GasLab is able to produce a flow of up to 10 L/min of complex trace gas mixtures diluted in dry or humidified zero air containing from 10 ppb to 100 ppm of each solute. However, for the goals of this study, pure substances were additionally diluted (1:2000–1:3000) with distilled water prior to evaporation in order to reduce the resulting concentration levels. Effectively, humid gas mixtures (100% RH at 37°C) with volume fractions ranging from approximately 0.04 to 350 ppb were used during calibration and validation. The calibration mixtures were sampled and analyzed using identical conditions as in the case of head-space measurements of cell cultures and blanks (i.e. volume of 200 ml at a flow rate of 10 ml/min at 37°C).

### Cell cultivation

**Figure 1:**
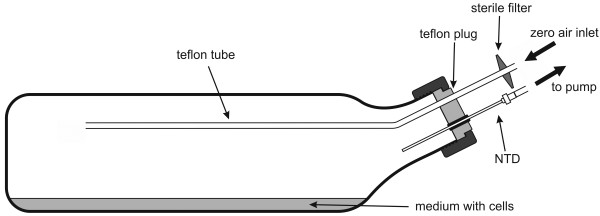
Cultivation/measurement bottle.

The epithelial hepatocellular carcinoma cell line HepG2 was used during the *in vitro* experiments. The cells were grown in Dulbecco's Modified Eagle high glucose (4.5 g/L) medium DMEM (PAA Laboratories, Pasching, Austria) containing 10% fetal calf serum (FCS) (PAA Laboratories, Pasching, Austria), 200 mM L-glutamine and penicillin (100 U/ml) (PAA Laboratories, Pasching, Austria), streptomycin (100 μg/ml) (PAA Laboratories, Pasching, Austria) in a conventional incubator at 37°C in a humidified atmosphere containing 92.5% air and 7.5% CO_2_. Prior to the measurements trypsinized cells were inoculated in 100 mL of phenol red free medium (DMEM high glucose, PAA Laboratories, Pasching, Austria) in special glass bottles (Ruprechter, Austria). The cultivation/measurement bottles had diameters of 21 cm × 5.5 cm × 11.5 cm (1000 ml nominal volume) and were closed with a Teflon plug (see Figure 1). Each Teflon plug was equipped with a rubber septum enabling the insertion of the needle trap devices into the headspace of the bottle and the Teflon tube being the inlet of the zero air stream. To provide proper mixing of the headspace during sampling the inner end of the Teflon tube protruded 15–17 cm from the plug into the headspace volume, whereas the outer end was equipped with a sterile filter. The bottom area of the bottles (approximately 240 cm^2^) was coated with Poly-Lysin (Sigma Aldrich, USA) and the caps were slightly loosened to allow ventilation during proliferation. Since the fresh medium was found to contain a number of volatile organic compounds exhibiting very high concentration levels, which impeded the detection and identification of volatiles released by the cells of interest, each new medium portion was flushed for 2–3 days with humidified high purity zero air produced by the GasLab generator at the flow rate of 100 ml/min. Such a treatment reduced the medium VOCs abundances approximately by a factor of 5–8. One day after subcultivation the culture media were changed and the bottles were sealed to facilitate the accumulation of species released by the cells and to avoid contamination by the ambient atmosphere. The analyses of the headspace were performed after 24h. Cell viability counts (trypan blue exclusion method) were performed immediately after the GC-MS analyses. In total 6 cultivation experiments have been performed.

In addition to the experiments involving cells cultures, blank (control) experiments were performed in parallel. These blank experiments followed the same protocol as mentioned above, however, without the addition of cells into the measurement bottles. An effort was made to always use the same flushed medium in blanks and corresponding cell cultures. The reproducibility of such a protocol was checked by a comparison of head-space levels of compounds under study (consumed ones) in five cultivation bottles containing the same medium after 24 hours of simulated cultivation. The calculated RSDs were smaller than 10%.

### NTD extraction procedure and chromatographic analysis

Volatile compounds were pre-concentrated manually using three-bed side-hole 23-gauge stainless steel needle trap devices (NTD) (PAS Technology, Germany) [54-56]. All needles were Silcosteel-treated and their sorbent beds consisted of 1 cm of Tenax TA (80/100 mesh), 1 cm of Carbopack X (60/80 mesh) and 1 cm of Carboxen 1000 (60/80 mesh). Prior to the first use all NTDs were pre-conditioned at 290°C by flushing them with high-purity nitrogen (6.0 – 99.9999%) for 4 h. Their re-conditioning was performed directly before sampling at the same temperature, however, with shorter flushing times of 10 minutes. Since NTDs were found to exhibit relatively huge differences with respect to the extraction efficiency (deviations of up to 70%, even when originating from the same production lot) the NTDs used during experiments were pre-selected according to the requirement that their inter-needle variability should be below 10%.

NTD trapping of headspace constituents was accomplished dynamically by inserting the NTD through a rubber septum into the headspace of the bottle and drawing 200 mL of sample at a steady flow rate of 10 mL/min at 37°C. This was done with the help of a membrane pump (Vacuubrand, Germany) and a mass flow controller (RED-Y, Burde Co. GmbH, Austria). Consequently, no transfer line had to be installed between the headspace sample and the needle trap. To maintain a constant pressure during sampling high purity zero air was continuously introduced into the bottle at a flow equal to the sampling flow. Following extraction the NTD was manually introduced into the inlet of the gas chromatograph where the compounds of interest were thermally desorbed at 290°C in a splitless mode (1 min).

Chromatographic analyses were performed using an Agilent 7890A/5975C GC-MS system (Agilent, USA). During NTD desorption, the split/splitless inlet operated in the splitless mode (1 min), followed by a split mode at ratio 1:20. The volatiles of interest were separated using a PoraBond Q column (25 m × 0.32 mm, film thickness 5 μm, Varian, USA) working in a constant flow mode (helium at 1.5 mL/min). The column temperature program was as follows: 40°C for 5 min, increase to 260°C at a rate of 7°C/min, followed by a constant temperature phase at 260°C for 6 min. The mass spectrometer worked in a SCAN mode with an associated m/z range set from 20 to 200. The quadrupole, ion source, and transfer line temperatures were kept at 150°C, 230°C, and 280°C, respectively.

The identification of compounds was performed in two steps. At first, the peak spectrum was checked against the NIST mass spectral library. Next, the NIST identification was confirmed by comparing the respective retention times with retention times obtained on the basis of standard mixtures prepared from pure compounds. Peak integration was based on extracted ion chromatograms. The retention times of the investigated compounds for the applied chromatographic parameters as well as the ions used for the integration are presented in Table 1.

## Competing interests

The authors declare that they have no competing interests.

## Authors’ contributions

PM contributed to the design of the study, developed the GC-MS measurement protocol, performed GC-MS analyses, calibration and validation measurements, data processing and interpretation, and wrote the draft of the manuscript. AS performed the cell culture experiments and revised the manuscript. AA and JT designed the study, supervised the experiments, revised and approved the manuscript. JK and KU participated in data analysis and interpretation, and revised the manuscript. All authors read and approved the final manuscript.
